# Comparison and Characterization of Mutations Induced by Gamma-Ray and Carbon-Ion Irradiation in Rice (*Oryza sativa* L.) Using Whole-Genome Resequencing

**DOI:** 10.1534/g3.119.400555

**Published:** 2019-09-13

**Authors:** Feng Li, Akemi Shimizu, Takeshi Nishio, Nobuhiro Tsutsumi, Hiroshi Kato

**Affiliations:** * Radiation Breeding Division, Institute of Crop Science, National Agricultural and Food Research Organization (NARO), Hitachi-ohmiya, Ibaraki 319-2293, Japan; † Graduate School of Agricultural Science, Tohoku University, Sendai, Miyagi 980-0845, Japan; ‡ Graduate School of Agricultural and Life Sciences, The University of Tokyo, Tokyo 113-8657, Japan, and; § Genetic Resources Center, NARO, Tsukuba, Ibaraki 305-8602, Japan

**Keywords:** Mutation breeding, Gamma rays, Heavy ions, Whole genome resequencing, Rice

## Abstract

Gamma-rays are the most widely used mutagenic radiation in plant mutation breeding, but detailed characteristics of mutated DNA sequences have not been clarified sufficiently. In contrast, newly introduced physical mutagens, *e.g.*, heavy-ion beams, have attracted geneticists’ and breeders’ interest and many studies on their mutation efficiency and mutated DNA characteristics have been conducted. In this study, we characterized mutations induced by gamma rays and carbon(C)-ion beams in rice (*Oryza sativa* L.) mutant lines at M_5_ generation using whole-genome resequencing. On average, 57.0 single base substitutions (SBS), 17.7 deletions, and 5.9 insertions were detected in each gamma-ray-irradiated mutant, whereas 43.7 single SBS, 13.6 deletions, and 5.3 insertions were detected in each C-ion-irradiated mutant. The structural variation (SV) analysis detected 2.0 SVs (including large deletions or insertions, inversions, duplications, and reciprocal translocations) on average in each C-ion-irradiated mutant, while 0.6 SVs were detected on average in each gamma-ray-irradiated mutant. Furthermore, complex SVs presumably having at least two double-strand breaks (DSBs) were detected only in C-ion-irradiated mutants. In summary, gamma-ray irradiation tended to induce larger numbers of small mutations than C-ion irradiation, whereas complex SVs were considered to be the specific characteristics of the mutations induced by C-ion irradiation, which may be due to their different radiation properties. These results could contribute to the application of radiation mutagenesis to plant mutation breeding.

Since Muller (1927) provided the proof of mutation induction by X-rays in *Drosphila* and Stadler (1928) published the first results of mutation induction in crop plants, mutation breeding has been developed rapidly to become a useful method for crop improvement. Since the 1960s, gamma-ray mutagenesis has been the most commonly used method in plant mutation breeding. Among the 3,281 mutant cultivars officially registered in FAO/IAEA mutant variety database (http://mvgs.iaea.org), 1,600 were obtained by gamma-ray irradiation. In Japan, 60% of the mutant varieties have been developed by gamma-ray irradiation (Nakagawa and Kato 2017).

During the past two decades, heavy-ion irradiation has also been accepted as an efficient mutagenesis technology. Gamma rays and heavy-ion beams are both ionizing radiations which are capable of causing the release or capture of electrons (called ionizations) and directly disrupting the chemical bonds of molecules when they pass through matter (Lagoda 2012; Mba *et al.* 2012). The toxic effects of ionizing radiation arise through the production of reactive oxygen species (ROS) that damage all components of a cell (Lagoda 2012). DNA lesions caused by ionizing radiation include nucleotide base lesions (base lesions), DNA single-strand breaks (SSBs), and double-strand breaks (DSBs) (Lomax *et al.* 2013). Most of the SSBs can be repaired via DNA ligation, while DSBs are mainly repaired in two pathways, namely, homologous recombination (HR) and nonhomologous end joining (NHEJ). Although HR and NHEJ have been usually regarded as error-free and error-prone, respectively, recent studies have revealed that both have the capacity to give rise to base pair substitutions, indels (insertion/deletion), and chromosome rearrangements (Rodgers and McVey 2016).

Heavy-ion beams have unique properties, *i.e.*, a heavy ion has a mass and an electrical charge. The linear energy transfer (LET) of gamma rays is 0.2 keV·μm^–1^, whereas the LET of heavy-ion beams can be altered from 22.5 to 4000 keV·μm^–1^ by selecting the ion species or controlling its velocity (Kazama *et al.* 2017). The biological effects of high- and low-LET radiations have been compared using cultured mammalian cells in a large number of studies (Yatagai 2004; Hagiwara *et al.* 2019). When a cell is irradiated, high-LET radiation deposits high energy densely along the particle track, causing a great number of dynamic chromosomal aberrations, including chromosome breaks, dicentrics, translocations, and large-size indels, and, in contrast, low-LET radiation induces DNA damage with a mostly random distribution in the nucleus. Accumulating evidence in clinical studies has demonstrated that C-ion radiotherapy is effective for tumors compared to X-ray radiotherapy (Loeffler and Durante 2013).

The different characteristics of gamma rays and heavy-ion beams have attracted researchers’ attention to compare their mutagenic effects (Shikazono *et al.* 2003; Matuo *et al.* 2006; Yamaguchi *et al.* 2009; Yoshihara *et al.* 2013). Mutagenic effectiveness, calculated as the frequency of mutations (usually chlorophyll mutations) divided by irradiation dose, and mutagenic efficiency, calculated as the frequency of mutations divided by percentage of lethality, injury, or sterility (Ambavane *et al.* 2015), have been used for comparison of the mutagenic effects of different mutagens. Mutagenic effectiveness of heavy-ion beams is significantly higher than that of gamma rays due to their high-LET nature (Shikazono *et al.* 2003; Matuo *et al.* 2006; Yamaguchi *et al.* 2009). As for mutagenic efficiency, a widely-used index in plant mutation breeding, no clear conclusion has been drawn. Investigation of the frequencies of chlorophyll mutants has revealed that mutation frequencies in M_2_ populations produced by heavy-ion irradiation are higher than those by gamma-ray irradiation under 70% or 90% shoulder doses of the survival curves in rice (*Oryza sativa* L.) (Yamaguchi *et al.* 2009). However, no significant differences were observed in mutation frequencies induced by C-ion and gamma-ray irradiations in *Arabidopsis* under an 80% shoulder dose of the survival curves (Yoshihara *et al.* 2013; Yoshihara *et al.* 2010). As for DNA mutation characteristics, it has been revealed that both types of radiation mostly induce large deletions (>80 kbp) using a pollen-irradiation method, which is expected to capture the most of all mutations (Naito *et al.* 2005). Irradiating dry seed of *Arabidopsis*, Yoshihara *et al.* (2010) have revealed that C-ion irradiation tends to induce large indels of more than 3 basepairs and gamma-ray irradiation has a greater tendency to induce small indels of 1 or 2 basepairs.

Recently, with the rapid developments of genomics and next-generation sequencing (NGS) technology, exome sequencing and whole-genome resequencing (WGR) were employed to study the molecular characterization of mutations on a whole-genome level, and a novel understanding of the effects of mutagenic agents has been achieved (Belfield *et al.* 2012; Henry *et al.* 2014; Li *et al.* 2017; Li *et al.* 2016b; Shirasawa *et al.* 2016; Du *et al.* 2017; Kazama *et al.* 2017; Ichida *et al.* 2019). For example, fast-neutron irradiation of dry seeds has been traditionally considered to predominantly induce deletion mutations of 2-4 kb in size (Bruggemann *et al.* 1996; Li *et al.* 2001), and gamma-ray irradiation has been revealed to induce mostly deletions, particularly small deletions (Morita *et al.* 2009), but WGR revealed that fast neutrons and gamma rays induced a higher frequency of single base substitutions (SBS) than deletion mutations, and more single-base deletions than large deletions (Belfield *et al.* 2012; Li *et al.* 2017; Li *et al.* 2016b; Shirasawa *et al.* 2016). The characterizations of mutations were compared between C-ion (LET: 30 keV·μm^–1^) irradiation and Ar-ion (LET: 290 keV·μm^–1^) irradiation using *Arabidopsis* mutants in M_3_ generation derived from irradiated dry seeds, and the results have revealed that both types of radiations mainly induce small mutations including SBSs and small indels (<100 bp) (Kazama *et al.* 2017). Furthermore, Ar ions induce chromosomal rearrangements or large deletions (≥100 bp) more frequently than C ions, and, in contrast, C ions induce more SBS and small indels than Ar ions (Kazama *et al.* 2017). However, since the analyzed mutants in the above-mentioned studies belong to different species, and the analysis methods are different, the mutation characteristics induced by different kinds of radiations cannot be accurately compared.

In this study, we comprehensively characterized the mutation effects of gamma rays (250 Gy) and C-ion beams (LET 107 keV/μm^-1^) by WGR of seven M_5_ mutant lines for each type of radiation. The numbers of SBSs, small indels, and rearrangements were compared for these two different irradiations.

## Materials and Methods

### Materials

In a previous study (Yamaguchi *et al.* 2009) mainly conducted in our institute, *i.e.*, the Institute of Radiation Breeding (Present name: Radiation Breeding Division, Institute of Crop Science, NARO, Hitachi-ohmiya, Japan), hulled dry seeds of a rice cultivar ‘Hitomebore’ were irradiated with 220 MeV C ions (LET 107 keV/μm) at doses from 10 to 60 Gy by an AVF-cyclotron (Japan Atomic Energy Agency, Takasaki, Japan), and unhulled dry seeds were irradiated with gamma rays from 150 to 450 Gy with a dose rate of 10 Gy/h in the gamma room of our institute. The morphological mutants isolated in that study have been kept and self-pollinated to obtain M_6_ progeny. The dose at which the lethal rate of the M_1_ population is from 10 to 50% (LD_10_ to LD_50_) can be used as the optimum dose (Yamaguchi *et al.* 2009; Kodym *et al.* 2012). In our institute, the most frequently used dose for gamma-ray irradiation of rice is 250 Gy, which is equal to LD_30_ (Yamaguchi *et al.* 2009). LD_30_ for 220 MeV C ions has been estimated to be about 30 Gy (Yamaguchi *et al.* 2009). In the present study, seven mutant lines (HTM_G347, HTM_G348, HTM_G349, HTM_G351, HTM_G353, HTM_G354, and HTM_G355) produced by gamma-ray irradiation and seven mutant lines (HTM_I154, HTM_I218, HTM_I223, HTM_I224, HTM_I226, HTM_I227, and HTM_I257) produced by C-ion irradiation at LD_30_ were chosen for WGR. Their mutation phenotypes are summarized in Table S1.

### Whole-genome resequencing

Young leaves were collected from more than 20 seedlings of each M_6_ mutant line. This was done in an attempt to re-create the zygosity of alleles in the M_5_ parental plant. DNA extraction was conducted using DNeasy Plant Maxi Kit (Qiagen Inc., Valencia, USA) according to the manufacturer’s protocol. DNA libraries were prepared with the TruSeq DNA Sample Preparation Kit (Illumina Inc.), and paired-end (2×150 bp) sequencing was performed on Illumina HiSeq X Ten to determine genomic sequences with about 30-fold depth for each line.

### Bioinformatics analysis

After removing adapter sequences and contaminations, the raw paired-end reads were cleaned by removing low quality reads and unpaired reads using Trimmomatic (version 0.36) (Bolger *et al.* 2014) with the following parameters: LEADING:10, TRAILING:10, SLIDINGWINDOW:4:20, and MINLEN:36. The workflow of short read mapping and SNP/indel calling was based on the best practices for variant discovery analysis outlined by the Broad Institute (https://software.broadinstitute.org/gatk/best-practices/ ; https://gencore.bio.nyu.edu/variant-calling-pipeline/). Briefly, the clean reads were mapped to the rice reference genome (Nipponbare, IRGSP-1.0, http://rapdb.dna.affrc.go.jp) (Kawahara *et al.* 2013) using the mapping tool Borrows Wheeler Aligner (version 0.7.17) (Li and Durbin 2009) and indexed as BAM files using SAMtools (version 1.3.1) (Li *et al.* 2009). Next, duplicate fragments were marked and eliminated with MarkDuplicates tool in Picard-Tools (Version 2.7.1.0) (http://broadinstitute.github.io/picard/). SNP and small indel (<100bp) (hereafter called small mutation) calling was performed using the HaplotypeCaller tool in GATK (Version 3.7-0) (McKenna *et al.* 2010) after IndelRealigner and Base Quality Score Recalibration (BQSR) steps. SNPs were filtered using VariantFiltration with QD < 2.0, FS > 60.0, and MQ < 20.0. Indels were filtered using VariantFiltration with QD < 2.0, FS > 200.0, and SOR > 10.0. Detection of structural variation (SV), including large indel (>= 100 bp), duplication, transversion, and translocation, was performed using Pindel (Ye *et al.* 2009), BreakDancer (Chen *et al.* 2009), and Manta (Chen *et al.* 2016) using the default parameters and visually confirmed using Integrative Genomics Viewer (IGV) (Robinson *et al.* 2011). Both a simple SV derived from one DSB and a complicated SV involving more than two DSBs were counted as one SV.

To filter the background mutations and to reduce the false positives, the candidate mutations were further extracted with three criteria: (1) the read depth of the variant site was more than five and less than 100, (2) more than 30% of the reads supported the variant, (3) candidate mutations were detected in only one mutant line. Mutation rate was calculated as the average number of mutations per mutant divided by the average length (number of bases) of all genomic regions with at least 5× coverage.

Mutation annotation was performed based on the gene models of the ‘Nipponbare’ reference genome using SnpEff v4.2 (Cingolani *et al.* 2012). Mutations detected in mutants were plotted in the rice reference genome (Nipponbare, IRGSP-1.0,) using Circos software (Krzywinski *et al.* 2009).

### Verification of identified mutations

The specific primers for amplifying 94 randomly selected small mutation sites and all the SV sites were designed by Primer3 program (http://bioinfo.ut.ee/primer3-0.4.0/). Polymerase chain reaction (PCR) was conducted in a reaction mixture of 10 μl, comprising 10 ng genomic DNA as a PCR template, 0.5 μM of primers, 0.25 U of PrimeSTAR GXL DNA Polymerase (Takara, Kusatsu, Japan), 1× PrimeSTAR GXL Buffer, and 200 μM of dNTPs. The PCR conditions were as follows: Pre-incubation at 98° for 1 min followed by 35 cycles of 10 s denaturation at 98°, 15 s annealing at 60°, 20 s extension at 68°, and final extension at 68° for 2 min. The presence of PCR products was confirmed by analyzing the products on a 1.5% agarose gel. Each PCR product appearing as a single band on the agarose gel was cleaned up using ExoSAP-IT Express PCR Cleanup Reagent (Thermo Fisher Scientific, Waltham, MA, USA) and sequenced on a 3730xl Genetic Analyzer (Applied Biosystems, Foster City, CA, USA) after a sequencing reaction using the BigDye Terminator V3.1 cycle sequencing kit (Applied Biosystems). Zygosity assessment was based on the Sanger sequencing chromatogram.

### Statistical analysis

The number and proportion of each category of mutations induced by gamma rays were compared with those induced by C-ion beams using Student’s *t*-tests and Fisher’s Exact, respectively, in R Statistical Software (version 3.2.2; R Foundation for Statistical Computing, Vienna, Austria).

### Data availability

All NGS data files are available in the DDBJ Sequenced Read Archive under the accession numbers from DRA008194 to DRA008207. Supplemental material available at FigShare: https://doi.org/10.25387/g3.7998425.

## Results

### Detection of mutations by whole-genome resequencing

Seven mutant lines obtained by gamma-ray irradiation and seven mutant lines obtained by C-ion irradiation were subjected to whole-genome resequencing using the NGS technologies. More than 57 million paired-end (2×150 bp) reads per sample were obtained (Table S2). After removing the low quality, unpaired, and duplicated reads, more than 81.6% of the clean reads were mapped to the ‘Nipponbare’ reference genome. The average coverages were 15.7-23.2 times and 94.0% of the genome on average had at least 5× coverage.

Candidates for small mutations were detected by the GATK software based on the best practices for variant discovery analysis and using strict extraction parameters as described in Materials and Methods. To understand whether these parameters effectively reduced false positives, 94 randomly selected mutations were subject to validation by Sanger sequencing, of which 69 were successfully sequenced. The concordance between NGS and Sanger sequencing mainly depended on the variant allele frequency, *i.e.*, percentage of sequencing reads supporting the variant allele (Table S3). Approximately 83.3% (n = 12) were identified as NGS false-positives when the variant allele frequency was < 40%, and, conversely, 98.2% (n = 57) of NGS were concordant with Sanger sequencing when the variant allele frequency was ≥ 40%. Mu *et al.* (2016) also suggested that a conservative threshold of a variant allele frequency of > 40% was necessary for high-confidence NGS calls. We checked the zygosity of the mutations based on the chromatogram of Sanger sequencing, and found that all the mutations (n = 23) with variant allele frequency < 75.0% were heterozygous, 88.2% (n = 17) were homozygous when the variant allele frequency was ≥ 75.0% and < 100.0%, and 100.0% (n = 16) were homozygous when the variant allele frequency was equal to 100.0%. Therefore, those variants with a variant allele frequency of more than 75% were called homozygous and those between 40% and 75% were called heterozygous. In total, 1,047 small mutations were identified in the 14 mutant lines (Table S4).

SVs were detected by Pindel, BreakDancer and Manta, followed by confirmation using the IGV. In total, 18 homozygous SVs were identified (Table S5), of which 9 and 1 were specifically detected by Manta and Pindel, respectively, 8 were commonly detected by Manta and Pindel or BreakDancer, indicating that Manta is robust for detection of SVs. Almost all of the SVs were confirmed by agarose gel electrophoresis or Sanger sequencing, except that one breakpoint junction in HTM_I223 was not confirmed.

### Comparison of the number of mutations induced by gamma rays and C-ion beams

**Figure 1:**
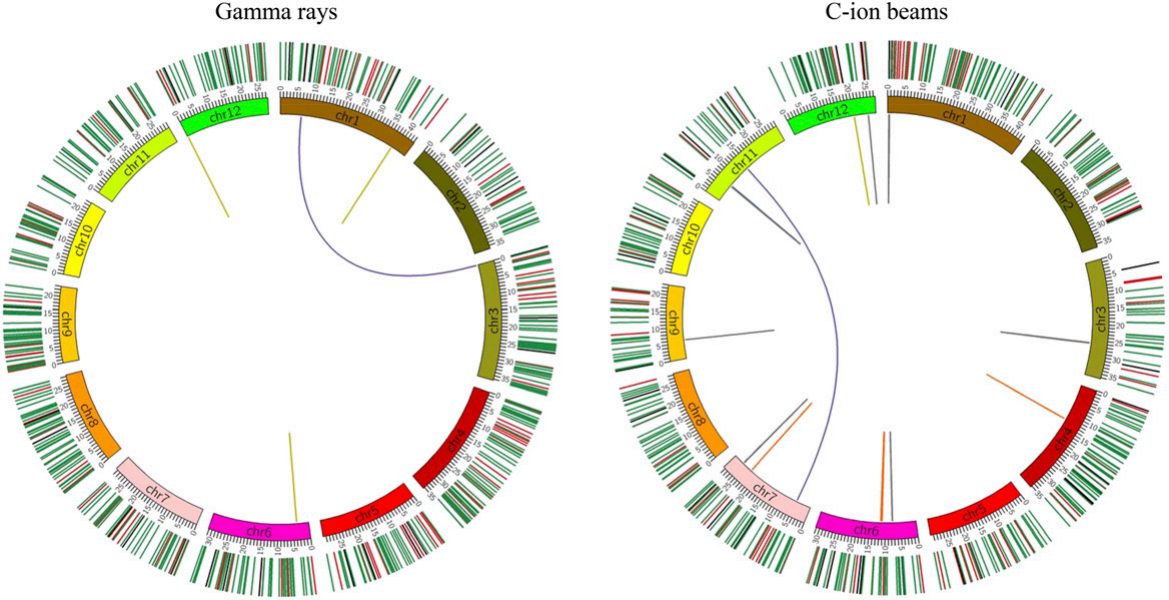
Circos diagrams illustrating differences between gamma ray- and C ion-induced mutations. All of the mutations in the seven mutants produced by gamma-ray and C-ion irradiation are plotted in the left and right diagrams, respectively. The structural variations (SVs) are plotted as lines into the interior of the circles. Orange, purple, gray, yellow and blue represent inversion, reciprocal translocation, large deletion (≥100 bp), large insertion (≥100 bp), and duplication, respectively. Small mutations are indicated by short lines on the exterior of the circles. Green, red and black represent single base substitutions (SBSs), small deletions (<100 bp), and small insertions (<100 bp), respectively.

A schematic diagram representing all the induced mutations is shown in Figure 1. As a result, 57.0 SBSs, 17.7 deletions, and 5.9 insertions on average were detected in each gamma-ray-irradiated mutant (Table 1). On the other hand, 43.7 SBSs, 13.6 deletions, and 5.3 insertions were detected in each C-ion-irradiated mutant. The SV analysis detected an average of 0.6 SVs in each gamma-ray-irradiated mutant, but an average of 2.0 in each C-ion-irradiated mutant. Statistically significant differences in the number of SBS were observed between gamma-ray and C-ion irradiations (*P* < 0.01; two side Student’s *t*-test), but not in the number of the other types of mutations. Given the length of all genomic regions with at least 5× coverage (351.4 Mb), the mutation rates of gamma-ray-irradiated and C-ion-irradiated mutants were estimated to be 23.1 ± 1.5 and 18.3 ± 3.4 (×10^−8^/bp), respectively, showing significant difference (*P* < 0.05; two side Student’s *t*-test). The theoretically expected ratio of homozygous mutations was 85.0%, because the bulked M_6_ plants, which represent the mutations of their M_5_ progenitor, were used in this study. The actual ratios of homozygosity for the gamma-ray-irradiated and C-ion-irradiated mutants were 86.7 ± 5.2 and 79.2 ± 4.7% (Table S6), respectively, neither of which were significantly different from the expected ratio of 85.0% (*P* > 0.05; chi-square test). According to the number of homozygous mutations, we estimated the total mutations from the M_1_ to the M_3_ generation (Table S6). For example, the total numbers of mutations in a whole genome of a gamma-ray-irradiated M_2_ mutant and a C-ion-irradiated M_2_ mutant were estimated to be 112.7 ± 10.4 and 79.3 ± 13.9, and the mutation rate to be 32.1 ± 3.0 and 23.5 ± 4.0 (×10^−8^/bp), respectively.

**Table 1:** Numbers of mutations induced by gamma rays and C-ion beams, respectively

Radiation type	Mutant ID	SBS	DEL	INS	SV	Total
Gamma rays	HTM_G347	60	19	7	2	88
	HTM_G348	56	18	7	0	81
	HTM_G349	57	27	5	0	89
	HTM_G351	59	10	3	2	74
	HTM_G353	52	17	7	0	76
	HTM_G354	61	17	5	0	83
	HTM_G355	54	16	7	0	77
	Average	57.0 ± 3.0 **	17.7 ± 4.7	5.9 ± 1.5	0.6 ± 0.9	81.1 ± 5.4 *
	Ratio (%)	70.4 ± 4.3	21.6 ± 4.3	7.2 ± 1.7	0.7 ± 1.1	100 ± 0.0
	Mutation rate in M5 (×10^−8^/bp)	16.2 ± 0.9	5.0 ± 1.3	1.7 ± 0.4	0.2 ± 0.3	23.1 ± 1.5 *
C-ion beams	HTMI-154	44	13	4	2	63
	HTMI-218	45	9	10	3	67
	HTMI-223	58	21	1	5	85
	HTMI-224	36	12	3	2	53
	HTMI-226	40	18	9	1	68
	HTMI-227	47	10	6	1	64
	HTM-I257	36	12	4	0	52
	Average	43.7 ± 7.1	13.6 ± 4.0	5.3 ± 3.0	2.0 ± 1.5	64.6 ± 10.2
	Ratio (%)	67.8 ± 3.9	20.9 ± 4.1	8.3 ± 4.1	2.9 ± 1.7 *	100 ± 0.0
	Mutation rate in M5 (×10^−8^/bp)	12.4 ± 2.0	3.9 ± 1.1	1.5 ± 0.9	0.6 ± 0.4	18.3 ± 3.4

SBS, single base substitution; DEL, deletion; INS, insertion; SV, structural variation.

** and * denote a significant difference between two radiation types at the 1 and 5% level, respectively, by Student's *t*-test.

### Comparison of the characteristics of small mutations induced by gamma rays and C-ion beams

**Figure 2:**
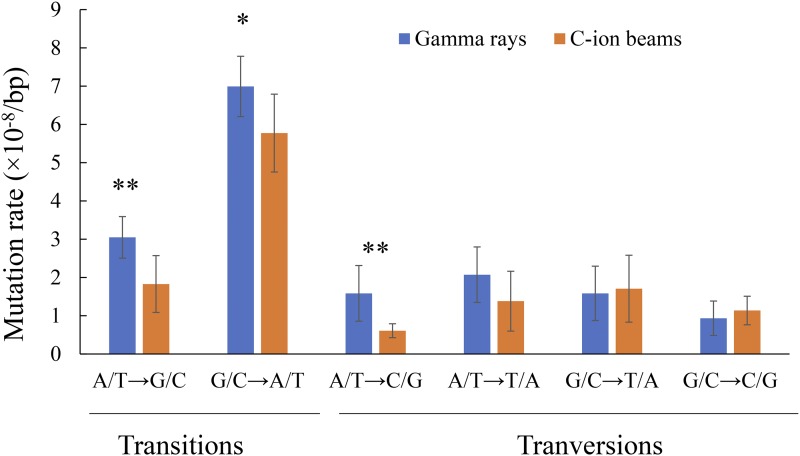
Mutation rate of different categories of SBSs induced by gamma-ray irradiation and C-ion irradiation. Asterisks indicate a significant difference between the two types of irradiation (*t*-test, * *P* < 0.05 and ** *P* < 0.01).

Exposures to gamma rays and C-ion beams both induced a relatively high frequency of SBSs, approximately 70.0% of the total mutations (Table 1). SBSs can be transitions (purine to purine; pyrimidine to pyrimidine) or transversions (purine to pyrimidine; pyrimidine to purine), and the SBSs were classified into six categories: A/T to G/G, G/C to A/T, A/T to C/G, A/T to T/A, G/C to T/A, and G/C to C/G (Figure S1). The proportion of each category in the gamma-ray-irradiated mutants and the C-ion-irradiated mutants were not significantly different (*P* > 0.05; Fisher’s exact test). The transition/transversion ratios of both gamma-ray irradiation and C-ion irradiation were near 1.6, which is comparable to the 1.4 of fast-neutron irradiation (Li *et al.* 2017). Among the SBSs, G/C to A/T transition was the most frequent in both the gamma-ray- and C-ion-irradiated mutants (43.0 ± 3.4 and 46.7 ± 6.6%, respectively (Figure S1)), and their mutation rates were 7.0 ± 0.8 and 5.8 ± 1.0 (×10^−8^/bp) (Figure 2), respectively, showing a significant difference (*P* < 0.05; two side Student’s *t*-test). The mutation rates of A/T to G/C transition and A/T to C/G transversion in the gamma-ray-irradiated mutants were also significantly higher than those in the C-ion-irradiated mutants (*P* < 0.01; two side Student’s *t*-test).

**Figure 3:**
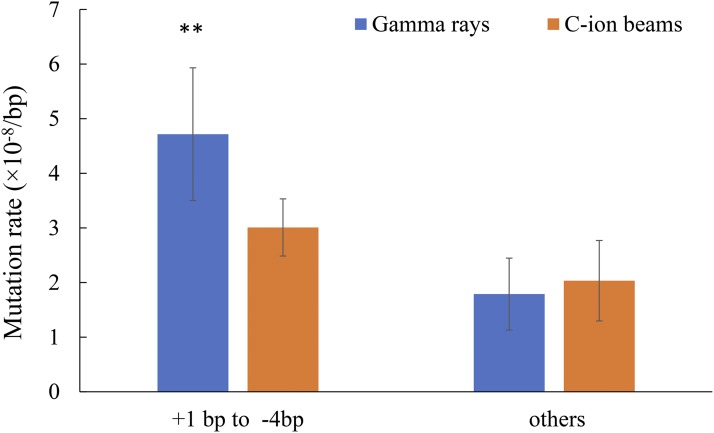
Mutation rate of different sizes of indels. +: insertion, -: deletion. Asterisks indicate a significant difference between gamma-ray irradiation and C-ion irradiation (*t*-test, ** *P* < 0.01).

The proportions of small DELs and INSs in the gamma-ray- and C-ion-irradiated mutants were approximately 21.0 and 8.0%, respectively (Table 1). Distributions of different sizes of indel mutations were similar for the mutants produced by gamma-ray irradiation and those by C-ion irradiation (Figure S2). Small indels from +1 bp to -4 bp were the common features in both the gamma ray- and C-ion-irradiated mutants (72.8 ± 7.4 and 60.4 ± 12.2%, respectively), and their mutation rates were 4.7 ± 1.2 and 3.0 ± 0.5 (×10^−8^/bp) (Figure 3), respectively, showing a significant difference (*P* < 0.01; two side Student’s *t*-test).

### Comparison of the characteristics of structural variations induced by gamma rays and C-ion beams

**Figure 4:**
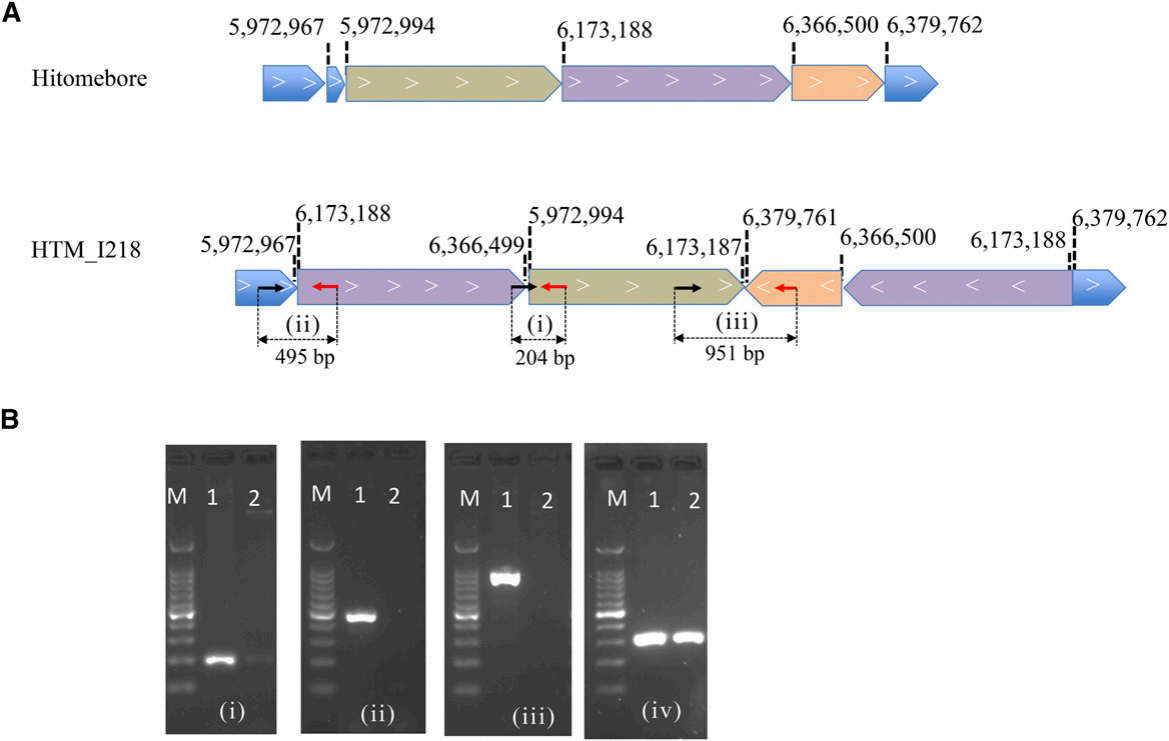
A complex structural variation induced by C-ion irradiation on chromosome chr11 of mutant ‘HTM_I218’. (A) Schematic representation of the genomic regions in ‘Hitomebore’ and ‘HTM_I218’. The positions of the breakpoints and directions of arrow-shaped boxes indicating 5′ to 3′ directions are based on the ‘Nipponbare’ genome sequence. Note that the diagram is not drawn to scale. To validate the SV, primer pairs (i): I218-SV1_f9 and I218-SV1_r9, (ii): I218-SV1_f4 and I218-SV1_r4, and (iii): I218-SV1_f2 and I218-SV1_r2 for PCR (Table S7) were designed. Arrows indicate the primer positions, and the predicted PCR product sizes are shown at the lower part. (B) Agarose gel electrophoresis of the amplification products obtained after PCR. The numbers (i), (ii), and (iii) of electrophoresis figures correspond to those in (A), and (iv) is the positive control for PCR using a primer pair control_f and control_r that are specific to a different genomic region without any mutations (Table S7). Lane M, 100 bp DNA ladder size marker; lane 1, ‘HTM_I218’ and lane 2, ‘Hitomebore’.

Large indels (>100bp), duplications, reciprocal translocations, and inversions were detected in the C-ion-irradiated mutants (Table S5). Furthermore, we detected one complicated SV involving multiple events of one duplication, one inversion, and one large deletion that occurred in chromosome 11 of HTM-I218 (Figure 4), and one complicated SV involving one inversion and one large deletion in chromosome 3 of HTM-I223 (Figure S3). Only large insertions and reciprocal translocations were detected in the gamma-ray-irradiated mutants (Table S5), *e.g.*, HTM-G347 (Figure S4). No other types of SVs were detected in the gamma-ray-irradiated mutants, which is considered to be due to the limited number of mutants used in this study. In previous studies, it has been reported that gamma-ray irradiation can induce large deletions, duplications, and inversions (Morita *et al.* 2009; Mase *et al.* 2014; Yuan *et al.* 2014; Li *et al.* 2019). The proportions of SVs in the gamma-ray- and C-ion-irradiated mutants were 0.7 ± 1.1 and 2.9 ± 1.7%, respectively, showing significant differences (Table 1) (*P* < 0.05; two side Student’s *t*-test). The proportion of SVs in the C-ion-irradiated mutants in this study was comparable with that in the C-ion-irradiated *Arabidopsis* mutant in the M_2_ generation (3.8%) (Kazama *et al.* 2017).

### Comparison of the number of mutated genes induced by gamma rays and C-ion beams

**Figure 5:**
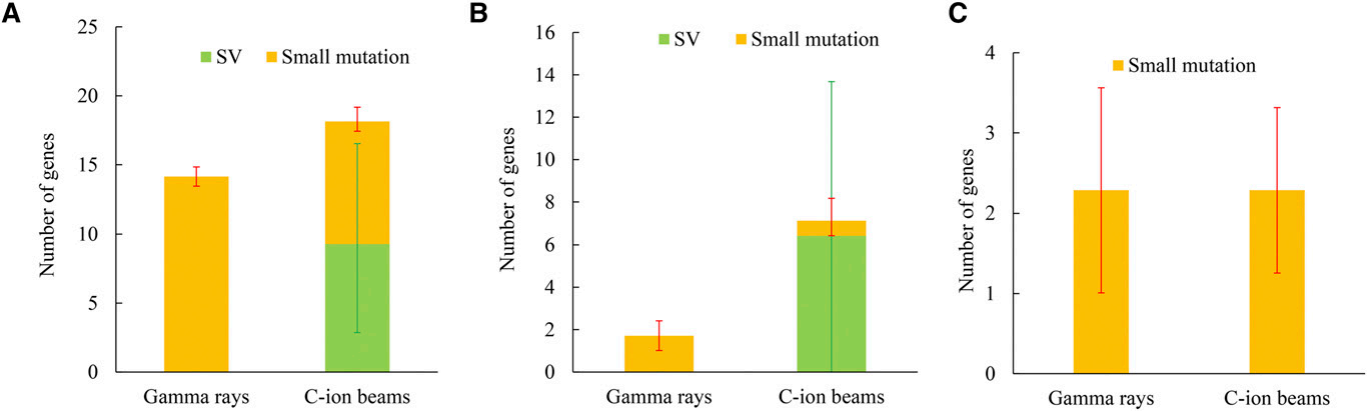
Numbers of affected genes per mutant irradiated by gamma rays and C-ion beams. (A) The number of all affected genes per line, orange: small mutation, green: SV. (B) The number of high-impacted genes per line. (C) The number of moderate-impacted genes per line.

The effects of the mutations on gene function were assessed using SnpEff (Cingolani *et al.* 2012). The mutations located in the gene region from 5′ UTR to 3′ UTR were considered to have some effects on gene function. On average, 14.1 ± 4.3 and 18.1 ± 10.5 genes were affected in each gamma-ray- and C-ion-irradiated mutant, respectively (Figure 5a), and no significant differences were observed between them (*P* > 0.05; two side Student’s *t*-test). It should be noted that the large insertions or translocations that occurred in HTM_G347 and HTM_G351 were out of genic regions, whereas the large deletions in HTM_I223, HTM_I154, and HTM_I224 caused 8, 21 and 12 genes to be truncated, respectively. Furthermore, a large duplication in HTM_I218 affected 20 genes. Except for the SVs, the small mutations induced by gamma rays and C-ion beams affected 14.1 ± 4.3 and 8.9 ± 3.2 genes, respectively, showing significant differences (*P* < 0.05; two side Student’s *t*-test).

The nonsense mutations, splice-site loss, and small indels causing frameshift, as well as large indels resulting in gene truncation, can be considered to have a high impact on a gene. Due to the large deletions in the C-ion-irradiated mutants, the number of highly-impacted genes were 7.1 ± 6.9, of which only 0.7 ± 1.0 were caused by small mutations (Figure 5b). In each gamma-ray-irradiated mutant, the number of highly-impacted genes were 1.7 ± 0.7, which were all caused by small mutations. No significant differences (*P* > 0.05; two side Student’s *t*-test) between gamma-ray- and C-ion-irradiated mutants were observed in the number of highly-impacted genes. The missense mutations and inframe deletions (lengths equal to a multiple of 3) were considered to have a moderate impact on a gene. The average numbers of moderately-impacted genes caused by small mutations in each gamma-ray- and C-ion-irradiated mutant were both near 2.3 (Figure 5c).

## Discussion

In this paper, we present a direct comparison between DNA mutations induced by gamma-ray irradiation and those by C-ion irradiation in rice, which were analyzed by whole-genome resequencing. Following irradiation of dry seeds at the optimum doses, as a whole, the total number of mutations in the M_5_ mutants generated by gamma-ray irradiation was significantly higher than that by C-ion irradiation (Table 1). The mutation rates in the genome of each M_2_ plant produced by gamma-ray and C-ion irradiations were estimated to be 32 and 24 (×10^−8^/bp), respectively (Table S6). It should be noted that these estimated mutation rates are considered to be slightly lower than the actual values, since some SVs causing lethality could not be inherited homozygously, although the percentage of SVs in the total number of mutations is very low in the M_2_ generation (Table 1) (Kazama *et al.* 2017). Using a TILLING method, Sato *et al.* (2006) have estimated the mutation rate induced by gamma-ray irradiation to be 12 (×10^−8^/bp) per M_2_ rice mutant. Li *et al.* (2017) have sequenced 1,504 fast neutron-irradiated rice mutants mainly in the M_2_ generation, and estimated the mutation rate to be 16 (×10^−8^/bp). The mutation rates obtained in the present study were higher than those estimated by these previous studies, probably due to the differences in irradiation conditions. More recently, Ichida *et al.* (2019) conducted whole-exome sequencing of unselected 110 M_2_ mutants obtained from dry rice seeds irradiated with 150 Gy of carbon ions (135 MeV/u, LET: 30 keV/μm). The mutation rate in an M_2_ mutant plant can be estimated to be 20 (×10^−8^/bp) (Ichida *et al.* 2019), which is close to our result. In *Arabidopsis*, mutation rates induced by fast-neutron and C-ion irradiations have been estimated to be 36 (×10^−8^/bp) (Belfield *et al.* 2012) and 43 (×10^−8^/bp) (Kazama *et al.* 2017). The higher mutation rate in *Arabidopsis* than that in rice might be due to species-specific differences in DSB repair (Kirik *et al.* 2000).

It should be noted that the type and frequency of detected mutation also depends on tissue type and method of analysis. Li *et al.* (2016b) have estimated that gamma-ray irradiation of rice seeds results in mutations at frequencies of 7.5 to 9.8 (×10^−6^/bp) in six M_2_ rice plants, which is over 10 times higher than our and other studies (Belfield *et al.* 2012; Li *et al.* 2016a; Li *et al.* 2017; Shirasawa *et al.* 2016; Du *et al.* 2017; Kazama *et al.* 2017; Hase *et al.* 2018; Ichida *et al.* 2019). This inconsistent result might be due to the high false positive rate in the study of Li *et al.* (2016b). Henry *et al.* (2015) analyzed over 500 F_1_ seedlings produced by pollinating *Populus deltoides* with gamma-irradiated *Populus nigra* pollen, and revealed that half of the samples exhibited at least one large indel with an average size of 5.8 Mb, while few point mutations were detected. These results confirm that irradiation of dry pollen mainly induces large deletions (Naito *et al.* 2005), whereas the majority of those large indels cannot be transmitted to progeny in seed-propagated diploid plant. More intrinsic differences in the characteristics between mutations induced by gamma-rays and those by carbon-ion beams would be revealed by using the pollen-irradiation method and NGS technologies. In the present study, we mainly discuss the characteristics of mutations in the mutants derived from irradiated dry seeds.

Considering the mutagenic effectiveness based on DNA mutation frequency, the mutagenic effectiveness of gamma rays (250 Gy) is 1.3 (×10^−9^/bp/Gy), which is lower than that of C ions (30 Gy), 8.0 (×10^−9^/bp/Gy), in the present study. This result is consistent with previous studies based on phenotypic mutation frequency (Shikazono *et al.* 2003; Matuo *et al.* 2006; Yamaguchi *et al.* 2009). It should be noted that mutagenic effectiveness is not important in mutation breeding, since irradiation dosage is easily adjusted. In contrast, mutagenic efficiency is widely used. Since the lethal rates of these two types of irradiation are both near 30%, as mentioned earlier, the mutagenic efficiency of gamma rays is higher than that of C ions, just as their DNA mutation frequencies are. However, the mutation frequency or mutagenic efficiency based on chlorophyll mutation show a contradictory result (Yamaguchi *et al.* 2009). Possible reasons for this discrepancy are discussed later.

The difference in the total number of mutations in the whole genome between gamma rays and C-ion beams is mainly attributed to the nucleotide base transition, 1-bp insertion, and < 4-bp deletions (Figures 2 and 3). Yoshihara *et al.* (2010; 2013) have also revealed that gamma rays tend to induce more transition and small deletions less than 2 bp. These results indicate that gamma-ray irradiation induces more minor modifications or DNA damage on DNA strands than ion-beam irradiation. The present study cannot confirm whether or not there are significant differences between the number of SVs induced by gamma-ray irradiation or C-ion irradiation due to their low frequencies and a small sample size. Furthermore, M_6_ plants were used in the present study, and, therefore, some mutations induced in M_1_ that are involved in gamete viability could not be inherited homozygously, as reported by Naito *et al.* (2005) and Kazama *et al.* (2017). We detected two complex SVs involving at least two or three DSB events in the C-ion-irradiated mutants HTM_I218 (Figure 4) and HTM_I223, respectively (Figure S3). These clustered DSBs may have been induced along the particle track in the cells following high-LET heavy-ion irradiation (Hagiwara *et al.* 2019). Complex SVs have also been detected in *Arabidopsis* and rice mutants irradiated by heavy ions and fast neutrons (which are also regarded as high-LET radiation), respectively (Li *et al.* 2016a; Kazama *et al.* 2017) . In contrast, the complex SVs have not been detected in gamma ray-irradiated mutants either in the present study or in previous studies (Naito *et al.* 2005; Morita *et al.* 2009; Henry *et al.* 2015; Datta *et al.* 2018; Li *et al.* 2019). Furthermore, the SVs induced by Ar-ion irradiation have been reported to be more complex than those induced by C-ion irradiation (Kazama *et al.* 2017). These results are consistent with previous findings that complex DNA lesions are the most representative hallmark of DNA damage induced by high-LET heavy-ion radiation (Hagiwara *et al.* 2019). However, some of the complex SVs and large deletions may not be advantageous for mutation breeding of seed-propagated diploid crops, since they may affect other phenotypes as well as the expected phenotype, resulting in accompanied undesirable traits. We also observed that the large deletions induced in three C-ion-irradiated mutants caused truncation of at least 8-21genes (Figure 5). In some previous studies (Naito *et al.* 2005; Yamaguchi *et al.* 2009), the higher frequencies of chlorophyll mutants induced by C-ion irradiation and gamma-ray irradiation in the M_2_ populations might be attributed to the complex SVs and large deletions. Furthermore, some SVs that cannot be inherited homozygously but exist heterozygously in early generations might also show observable phenotypes due to the role of gene dosage (Henry *et al.* 2015; Datta *et al.* 2018). If these SVs are excluded, the mutagenic efficiency of C-ion irradiation is not higher than that of gamma-ray irradiation. Furthermore, the operation of gamma-ray irradiation is simpler than that of heavy-ion irradiation and the cost of the former is lower than that of the latter. Therefore, it can be concluded that gamma-ray irradiation is more suitable for mutation breeding of seed-propagated diploid crops than heavy-ion irradiation.

Another widely used technique for mutagenesis, alkylating agents such as ethyl methanesulfonate (EMS), can cause an approximately ten times higher rate of mutation (mainly base substitutions from G/C to A/T) than radiations (Wu *et al.* 2005; Sato *et al.* 2006; Henry *et al.* 2014; Shirasawa *et al.* 2016). However, alkylating agents are strongly carcinogenic and cytotoxic, and there are health and safety risks when handling treated seeds even after careful washing. In contrast, there is no necessity for special post-treatment handling after gamma-ray irradiation, and it can be applied also to large seeds and growing plants having apical meristems covered by leaves. These advantages of gamma-ray irradiation might be the major reasons why most of the mutant cultivars have been obtained by gamma-ray irradiation.

As for vegetatively propagated or polyploid plants, SVs including large indels may be important for generating observable phenotypes and investigating the role of gene dosage (Henry *et al.* 2015; Datta *et al.* 2018). Almost all the mutations can be genetically fixed without meiosis and segregation in vegetatively propagated plants after irradiation. Although chimeric sectors exist in the irradiated plants due to mutagenesis of multicellular tissues, homogeneous mutants can be obtained by propagating the shoot tip in a plantlet for several generations (Datta *et al.* 2018). It would be interesting to compare the genome-scale characteristics of mutations induced by gamma-ray and carbon-ion irradiation in these crops in the future.

## References

[bib1] Ambavane A. R., Sawardekar S. V., Sawantdesai S. A., and Gokhale N. B., 2015 Studies on mutagenic effectiveness and efficiency of gamma rays and its effect on quantitative traits in finger millet (Eleusine coracana L. Gaertn). J Radiat Res Applied Sci 8: 120–125. 10.1016/j.jrras.2014.12.004

[bib2] Belfield E. J., Gan X., Mithani A., Brown C., Jiang C., 2012 Genome-wide analysis of mutations in mutant lineages selected following fast-neutron irradiation mutagenesis of *Arabidopsis thaliana*. Genome Res. 22: 1306–1315. 10.1101/gr.131474.111 22499668 PMC3396371

[bib3] Bolger A. M., Lohse M., and Usadel B., 2014 Trimmomatic: a flexible trimmer for Illumina sequence data. Bioinformatics 30: 2114–2120. 10.1093/bioinformatics/btu170 24695404 PMC4103590

[bib4] Bruggemann E., Handwerger K., Essex C., and Storz G., 1996 Analysis of fast neutron-generated mutants at the *Arabidopsis thaliana HY4* locus. Plant J. 10: 755–760. 10.1046/j.1365-313X.1996.10040755.x 8893551

[bib5] Chen K., Wallis J. W., McLellan M. D., Larson D. E., Kalicki J. M., 2009 BreakDancer: an algorithm for high-resolution mapping of genomic structural variation. Nat. Methods 6: 677–681. 10.1038/nmeth.1363 19668202 PMC3661775

[bib6] Chen X., Schulz-Trieglaff O., Shaw R., Barnes B., Schlesinger F., 2016 Manta: rapid detection of structural variants and indels for germline and cancer sequencing applications. Bioinformatics 32: 1220–1222. 10.1093/bioinformatics/btv710 26647377

[bib7] Cingolani P., Platts A., Wang L. L., Coon M., Nguyen T., 2012 A program for annotating and predicting the effects of single nucleotide polymorphisms, SnpEff: SNPs in the genome of Drosophila melanogaster strain w1118; iso-2; iso-3. Fly (Austin) 6: 80–92. 10.4161/fly.19695 22728672 PMC3679285

[bib8] Datta S., Jankowicz-Cieslak J., Nielen S., Ingelbrecht I., and Till B. J., 2018 Induction and recovery of copy number variation in banana through gamma irradiation and low-coverage whole-genome sequencing. Plant Biotechnol. J. 16: 1644–1653 10.1111/pbi.12901 PMC6097122 29476650

[bib9] Du Y., Luo S., Li X., Yang J., Cui T., 2017 Identification of substitutions and small insertion-deletions induced by carbon-ion beam irradiation in *Arabidopsis thaliana*. Front. Plant Sci. 8: 1851 10.3389/fpls.2017.01851 29163581 PMC5665000

[bib10] Hagiwara Y., Oike T., Niimi A., Yamauchi M., Sato H., 2019 Clustered DNA double-strand break formation and the repair pathway following heavy-ion irradiation. J. Radiat. Res. 60: 69–79. 10.1093/jrr/rry096 30476166 PMC6373698

[bib11] Hase Y., Satoh K., Kitamura S., and Oono Y., 2018 Physiological status of plant tissue affects the frequency and types of mutations induced by carbon-ion irradiation in *Arabidopsis*. Sci. Rep. 8: 1394 10.1038/s41598-018-19278-1 29362368 PMC5780457

[bib12] Henry I. M., Nagalakshmi U., Lieberman M. C., Ngo K. J., Krasileva K. V., 2014 Efficient genome-wide detection and cataloging of EMS-induced mutations using exome capture and next-generation sequencing. Plant Cell 26: 1382–1397. 10.1105/tpc.113.121590 24728647 PMC4036560

[bib13] Henry I. M., Zinkgraf M. S., Groover A. T., and Comai L., 2015 A System for Dosage-Based Functional Genomics in Poplar. Plant Cell 27: 2370–2383. 10.1105/tpc.15.00349 26320226 PMC4815095

[bib14] Ichida H., Morita R., Shirakawa Y., Hayashi Y., and Abe T., 2019 Targeted exome sequencing of unselected heavy-ion beam-irradiated populations reveals less-biased mutation characteristics in the rice genome. Plant J. 98: 301–314. 10.1111/tpj.14213 30584677 PMC6850588

[bib15] Kawahara Y., de la Bastide M., Hamilton J. P., Kanamori H., McCombie W. R., 2013 Improvement of the *Oryza sativa* Nipponbare reference genome using next generation sequence and optical map data. Rice (N. Y.) 6: 4 10.1186/1939-8433-6-4 24280374 PMC5395016

[bib16] Kazama Y., Ishii K., Hirano T., Wakana T., Yamada M., 2017 Different mutational function of low- and high-linear energy transfer heavy-ion irradiation demonstrated by whole-genome resequencing of *Arabidopsis* mutants. Plant J. 92: 1020–1030. 10.1111/tpj.13738 29024116

[bib17] Kirik A., Salomon S., and Puchta H., 2000 Species-specific double-strand break repair and genome evolution in plants. EMBO J. 19: 5562–5566. 10.1093/emboj/19.20.5562 11032823 PMC314016

[bib18] Kodym A., Afza R., Forster B. P., Ukai Y., Nakagawa H., 2012 Methodology for physical and chemical mutagenic treatments, pp. 169–180 in Plant Mutation Breeding and Biotechnology, edited by Shu Q. Y., Forster B. P. and Nakagawa H. Joint FAO/IAEA Division of nuclear Techniques in Food and Agriculture International Atomic Energy Agency, Vienna.

[bib19] Krzywinski M., Schein J., Birol I., Connors J., Gascoyne R., 2009 Circos: an information aesthetic for comparative genomics. Genome Res. 19: 1639–1645. 10.1101/gr.092759.109 19541911 PMC2752132

[bib20] Lagoda P. J. I., 2012 Effects of Radiation on Living Cells and Plants, pp. 123–134 in Plant Mutation Breeding and Biotechnology, edited by Shu Q. Y., Forster B. P. and Nakagawa H. Joint FAO/IAEA Division of nuclear Techniques in Food and Agriculture International Atomic Energy Agency, Vienna.

[bib21] Li F., Numa H., Hara N., Sentoku N., Ishii T., 2019 Identification of a locus for seed shattering in rice (*Oryza sativa* L.) by combining bulked segregant analysis with whole-genome sequencing. Mol. Breed. 39: 36 10.1007/s11032-019-0941-3

[bib22] Li G., Chern M., Jain R., Martin J. A., Schackwitz W. S., 2016 a Genome-wide sequencing of 41 rice (*Oryza sativa* L.) mutated lines reveals diverse mutations induced by fast-neutron irradiation. Mol. Plant 9: 1078–1081. 10.1016/j.molp.2016.03.009 27018389

[bib23] Li G., Jain R., Chern M., Pham N. T., Martin J. A., 2017 The sequences of 1504 mutants in the model rice variety Kitaake facilitate rapid functional genomic studies. Plant Cell 29: 1218–1231. 28576844 10.1105/tpc.17.00154 PMC5502455

[bib24] Li H., and Durbin R., 2009 Fast and accurate short read alignment with Burrows-Wheeler transform. Bioinformatics 25: 1754–1760. 10.1093/bioinformatics/btp324 19451168 PMC2705234

[bib25] Li H., Handsaker B., Wysoker A., Fennell T., Ruan J., 2009 The sequence alignment/map format and SAMtools. Bioinformatics 25: 2078–2079. 10.1093/bioinformatics/btp352 19505943 PMC2723002

[bib26] Li S., Zheng Y. C., Cui H. R., Fu H. W., Shu Q. Y., 2016 b Frequency and type of inheritable mutations induced by γ rays in rice as revealed by whole genome sequencing. J. Zhejiang Univ. Sci. B 17: 905–915. 10.1631/jzus.B1600125 27921396 PMC5172596

[bib27] Li X., Song Y. J., Century K., Straight S., Ronald P., 2001 A fast neutron deletion mutagenesis-based reverse genetics system for plants. Plant J. 27: 235–242. 10.1046/j.1365-313x.2001.01084.x 11532169

[bib28] Loeffler J. S., and Durante M., 2013 Charged particle therapy–optimization, challenges and future directions. Nat. Rev. Clin. Oncol. 10: 411–424. 10.1038/nrclinonc.2013.79 23689752

[bib29] Lomax M. E., Folkes L. K., and O’Neill P., 2013 Biological consequences of radiation-induced DNA damage: relevance to radiotherapy. Clin. Oncol. (R. Coll. Radiol.) 25: 578–585. 10.1016/j.clon.2013.06.007 23849504

[bib30] Mase N., Sawamura Y., Yamamoto T., Takada N., Nishio S., 2014 A segmental duplication encompassing S-haplotype triggers pollen-part self-compatibility in Japanese pear (*Pyrus pyrifolia*). Mol. Breed. 33: 117–128. 10.1007/s11032-013-9938-5 24482602 PMC3890579

[bib31] Matuo Y., Nishijima S., Hase Y., Sakamoto A., Tanaka A., 2006 Specificity of mutations induced by carbon ions in budding yeast *Saccharomyces cerevisiae*. Mutat. Res. 602: 7–13. 10.1016/j.mrfmmm.2006.07.001 16949109

[bib32] Mba C., Afza R., and Shub Q. Y., 2012 Mutagenic radiations: X-rays, ionizing particles and ultraviolet, pp. 83–90 in Plant Mutation Breeding and Biotechnology, edited by Shu Q. Y., Forster B. P. and Nakagawa H. Joint FAO/IAEA Division of nuclear Techniques in Food and Agriculture International Atomic Energy Agency, Vienna.

[bib33] McKenna A., Hanna M., Banks E., Sivachenko A., Cibulskis K., 2010 The Genome Analysis Toolkit: a MapReduce framework for analyzing next-generation DNA sequencing data. Genome Res. 20: 1297–1303. 10.1101/gr.107524.110 20644199 PMC2928508

[bib34] Morita R., Kusaba M., Iida S., Yamaguchi H., Nishio T., 2009 Molecular characterization of mutations induced by gamma irradiation in rice. Genes Genet. Syst. 84: 361–370. 10.1266/ggs.84.361 20154423

[bib35] Mu W. B., Lu H. M., Chen J., Li S. W., and Elliott A. M., 2016 Sanger confirmation is required to achieve optimal sensitivity and specificity in next-generation sequencing panel testing. J. Mol. Diagn. 18: 923–932. 10.1016/j.jmoldx.2016.07.006 27720647

[bib36] Muller H. J., 1927 Artificial transmutation of the gene. Science 66: 84–87. 10.1126/science.66.1699.84 17802387

[bib37] Naito K., Kusaba M., Shikazono N., Takano T., Tanaka A., 2005 Transmissible and nontransmissible mutations induced by irradiating *Arabidopsis thaliana* pollen with gamma-rays and carbon ions. Genetics 169: 881–889. 10.1534/genetics.104.033654 15371348 PMC1449103

[bib38] Nakagawa H., and Kato H., 2017 Induced mutations for food and energy security: Challenge of inducing unique mutants for new cultivars and molecular research. *Bull. NARO*. Crop Sci. 1: 22–124.

[bib39] Robinson J. T., Thorvaldsdottir H., Winckler W., Guttman M., Lander E. S., 2011 Integrative genomics viewer. Nat. Biotechnol. 29: 24–26. 10.1038/nbt.1754 21221095 PMC3346182

[bib40] Rodgers K., and McVey M., 2016 Error-prone repair of DNA double-strand breaks. J. Cell. Physiol. 231: 15–24. 10.1002/jcp.25053 26033759 PMC4586358

[bib41] Sato Y., Shirasawa K., Takahashi Y., Nishimura M., and Nishio T., 2006 Mutant selection from progeny of gamma-ray-irradiated rice by DNA heteroduplex cleavage using *Brassica* petiole extract. Breed. Sci. 56: 179–183. 10.1270/jsbbs.56.179

[bib42] Shikazono N., Yokota Y., Kitamura S., Suzuki C., Watanabe H., 2003 Mutation rate and novel tt mutants of *Arabidopsis thaliana* induced by carbon ions. Genetics 163: 1449–1455. 12702688 10.1093/genetics/163.4.1449 PMC1462525

[bib43] Shirasawa K., Hirakawa H., Nunome T., Tabata S., and Isobe S., 2016 Genome-wide survey of artificial mutations induced by ethyl methanesulfonate and gamma rays in tomato. Plant Biotechnol. J. 14: 51–60. 10.1111/pbi.12348 25689669 PMC5023996

[bib44] Stadler L. J., 1928 Genetic Effects of X-Rays in Maize. Proc. Natl. Acad. Sci. USA 14: 69–75. 10.1073/pnas.14.1.69 16587308 PMC1085350

[bib45] Wu J. L., Wu C., Lei C., Baraoidan M., Bordeos A., 2005 Chemical- and irradiation-induced mutants of indica rice IR64 for forward and reverse genetics. Plant Mol. Biol. 59: 85–97. 10.1007/s11103-004-5112-0 16217604

[bib46] Yamaguchi H., Hase Y., Tanaka A., Shikazono N., Degi K., 2009 Mutagenic effects of ion beam irradiation on rice. Breed. Sci. 59: 169–177. 10.1270/jsbbs.59.169

[bib47] Yatagai F., 2004 Mutations induced by heavy charged particles. Biol. Sci. Space 18: 224–234. 10.2187/bss.18.224 15858389

[bib48] Ye K., Schulz M. H., Long Q., Apweiler R., and Ning Z., 2009 Pindel: a pattern growth approach to detect break points of large deletions and medium sized insertions from paired-end short reads. Bioinformatics 25: 2865–2871. 10.1093/bioinformatics/btp394 19561018 PMC2781750

[bib49] Yoshihara R., Hase Y., Sato R., Takimoto K., and Narumi I., 2010 Mutational effects of different LET radiations in *rpsL* transgenic *Arabidopsis*. Int. J. Radiat. Biol. 86: 125–131. 10.3109/09553000903336826 20148698

[bib50] Yoshihara R., Nozawa S., Hase Y., Narumi I., Hidema J., 2013 Mutational effects of gamma-rays and carbon ion beams on *Arabidopsis* seedlings. J. Radiat. Res. (Tokyo) 54: 1050–1056. 10.1093/jrr/rrt074 23728320 PMC3823791

[bib51] Yuan L., Dou Y., Kianian S. F., Zhang C., and Holding D. R., 2014 Deletion mutagenesis identifies a haploinsufficient role for γ-zein in opaque2 endosperm modification. Plant Physiol. 164: 119–130. 10.1104/pp.113.230961 24214534 PMC3875793

